# Emission Intensity Readout of Ion-Selective Electrodes Operating under an Electrochemical Trigger

**DOI:** 10.1021/acs.analchem.1c00857

**Published:** 2021-07-15

**Authors:** Katarzyna Węgrzyn, Justyna Kalisz, Emilia Stelmach, Krzysztof Maksymiuk, Agata Michalska

**Affiliations:** Faculty of Chemistry, University of Warsaw, Pasteura 1, Warsaw 02-093, Poland

## Abstract

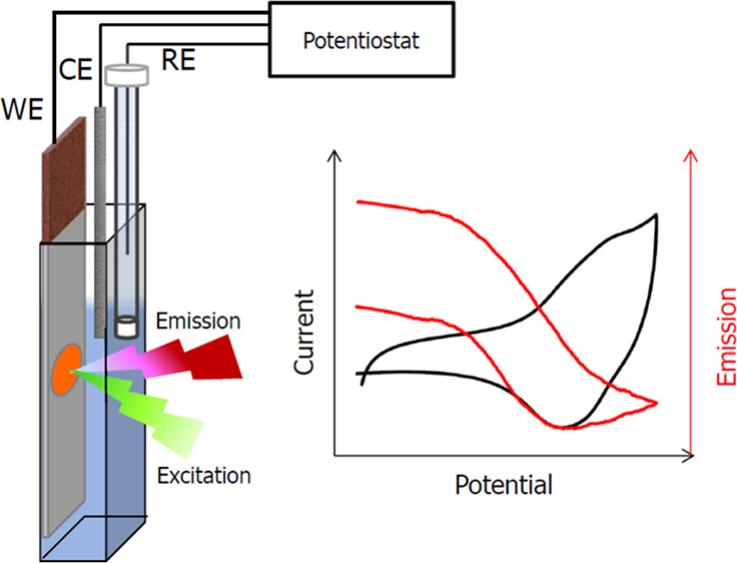

We report for the first time on in situ transduction of electrochemical responses of ion-selective electrodes, operating under non-zero-current conditions, to emission change signals. The proposed novel-type PVC-based membrane comprises a dispersed redox and emission active ion-to-electron transducer. The electrochemical trigger applied induces a redox process of the transducer, inducing ion exchange between the membrane and the solution, resulting also in change of its emission spectrum. It is shown that electrochemical signals recorded for ion-selective electrodes operating under voltammetric/coulometric conditions correlate with emission intensity changes recorded in the same experiments. Moreover, the proposed optical readout offers extended linear response range compared to electrical signals recorded in voltammetric or coulometric mode.

Ion-selective membranes (ISMs) were developed for potentiometric (open-circuit) sensors intended mostly for clinical/environmental applications. Nowadays, ISMs are also of interest for other applications, e.g., decentralized ion sensing for diagnostic or wearable applications^1^ and electrolyte-gated transistors.^2^ Due to the high selectivity offered, ISMs are attractive for sensors operating under controlled current or potential in voltammetric/coulometric/chronopotentiometric mode of “redox-inactive” ion selective sensing.^3−5^ For these applications, two layers of sensors are typically applied, with the ISM layer coated on an electroactive transducer. An electrochemical trigger induces a redox process in the transducer, ion exchange with the ISM, ion transport in the phase, and ion exchange on the ISM–sample interface. Ultimately, selective incorporation of analyte ions to the membrane phase results in analytical, electrochemical signals. Although potentiometric methods may offer low detection limits, the non-zero-current methods offer significant advantages for sensing but also reveal challenges due to the high resistive nature of ion-selective membranes. Alternatively, the optical readout mode herein proposed offers a possibility to overcome these issues, ultimately to offer a higher sensitivity or to extend an analytically useful range.

Voltammetry/coulometry using ion-selective membranes can be foreseen as a descendant of ion-transfer voltammetry at immiscible liquid interfaces.^6^ Different modes of controlled current/potential approaches offer different benefits. The voltammetric mode of ion-selective electrode application allowed the detection of perchlorate, potassium, or ammonium ions at a nanomolar concentration level.^7,8^ On the other hand, the chronopotentiometric approach is useful to control ion fluxes in the ISM, leading to a lower detection limit of the sensor.^5^ A significant increase in sensitivity is offered by the constant-potential coulometry approach.^4,9^ Extraction of ions from a thin solution layer using an ion-selective electrode operating in coulometric mode allows improving sensitivity and selectivity without the need for frequent recalibration of sensors.^10^ Electrochemical trigger-based sensing is also explored in ISM-based transistors using different configurations applied to the membrane on the gate^11^ or on the conducting polymer channel.^2^

Non-zero-current ISM applications typically require the presence of a relatively thin film to reduce the resistance of the system.^4,12^ Most often polyoctylthiophene (POT) – redox and optical active polymer is applied as transducer.^3^ In a neutral, semiconducting form, POT is characterized with bright emission, whereas for the oxidized polymer, emission is significantly quenched.^13^ This effect is observed for either films or nanoparticles^14^ (although emission spectra are different). The emission mode is highly sensitive to polymer redox state changes,^13,15^ offering the possibility of optical readout of processes occurring in systems operating under an electrochemical trigger, an approach yet not explored to our best knowledge.

Even under zero-current potentiometric conditions, the optical readout of ISM signals was considered only for chromoionophore-containing H^+^ sensors to study undesired coextraction of anions^16,17^ but not as an alternative readout mode. On the other hand, spectroelectrochemistry, understood as combining the optical readout with electrochemical trigger, was successfully applied, e.g., to study processes occurring in conducting polymers belonging to the polythiophene family or other optically active systems^15,18,19^ (in the absence of ISMs).

The novel idea of this work is to explore the emission change of POT corresponding to the redox transition of the polymer dispersed within the ISM forced by an applied trigger as a signal of an electrochemical reaction occurring (Figure 1). The application of a composite material as a membrane is attractive as (i) it allows elimination of spontaneous partition of POT to the membrane phase,^20^ offering full control of membrane composition. (ii) In consequence, effects related to interactions between POT and the ionophore/ion exchanger are controlled, too.^21^ (iii) A composite membrane with particulates of POT dispersed within the plasticized PVC matrix seems to be an attractive alternative also to assure a larger contact area between the conducting polymer and ion-selective membrane, which is proven as an important factor for sensors operating under coulometric conditions.^22^ We propose an emission readout of ion-selective sensors operating under non-zero-current conditions benefiting from a novel-type fluoroelectrochemical ion-selective membrane (FE-ISM). As a model system, potassium-selective sensors were studied.

**Figure 1:**
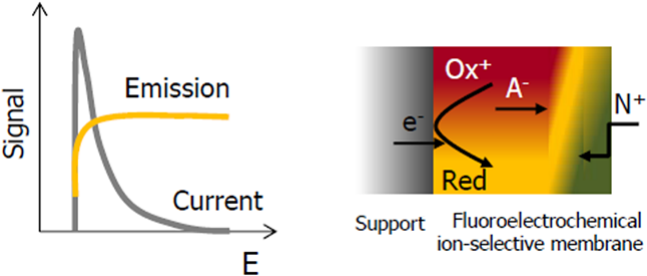
Schematic representation of the idea of fluoroelectrochemical experiments using ion-selective membranes.

## Experimental Section

### Reagents

Valinomycin, sodium tetrakis[3,5-bis(trifluoromethyl)phenyl]borate (NaTFPB), bis(2-ethylhexyl) sebacate (DOS), poly(vinyl chloride) (PVC), and tetrahydrofuran (THF) were obtained from Sigma Aldrich. Poly(3-octylthiophene-2,5-diyl) (POT) was obtained from Rieke Metals. Potassium chloride, sodium chloride, and K_4_[Fe(CN)_6_] were of analytical grade and were obtained from POCh (Gliwice, Poland). Ultrapure water with a resistivity of 18.2 MΩ cm (Milli-Q plus, Millipore) was used throughout the work.

### Preparation of Potassium-Selective FE-ISE Sensors

FE-ISE electrodes were obtained by drop-casting of a cocktail on the surface of carbon paper (Toray Carbon Paper PTFE-treated, Alfa Aesar, 200 μm thickness) (Figure S1). The surface area of the working electrode was limited to 0.137 cm^2^ by Teflon tape, and in turn, an electrical contact was provided by the use of a copper tape. The FE-ISE cocktail used contained (% by weight) 4.9% of POT, 2% of NaTFPB, 9.8% of valinomycin, 22% of PVC, and 61.3% of DOS. If not stated otherwise, 40 μL of the cocktail was applied per electrode, while for some experiments, thinner membranes were used and obtained by casting 15 μL of the cocktail. The estimated thickness of fluoroelectrochemical ion-selective membranes (by an IP54 digital micrometer 1–2″/25–50 mm) was 50 ± 2 μm and 78 ± 2 μm (*n* = 3, in both cases) for 15 or 40 μL of cocktail applied, respectively. A total of 46 mg membrane components were dissolved in 1 mL of THF. The mole ratio of membrane components was 10.9:1:3.8 for POT (mere units):NaTFPB:valinomycin. Before experiments, fluoroelectrochemical ion-selective membranes were conditioned for 1 h in a 10^–3^ M solution of KCl.

### Apparatus and Techniques

The fluorimetric and electrochemical measurements were performed using a spectrofluorimeter (Cary Eclipse) (Agilent Technologies) and potentiostat/galvanostat (CH Instruments model 760C) (Austin, TX, USA), respectively.

Fluoroelectrochemical ion-selective membranes were characterized by the following electrochemical techniques: cyclic voltammetry, chronoamperometry, and impedance spectroscopy and an optical technique, namely, fluorimetry. Simultaneous experiments are as follows: electrochemical and fluorimetric experiments were carried out at a disposable cuvette using a conventional three-electrode setup with Ag/AgCl (3 M KCl) as the reference electrode, Pt wire (1 mm diameter) as the counter electrode, and FE-ISE as the working electrode.

Emission spectra were recorded in the range from 600 to 800 nm after excitation at 550 nm; to observe intensity changes in time or for applied voltage, emission measurements at 720 nm were chosen. The excitation and emission slits were 10 nm, while the fluorimeter detector voltage was maintained at 750 V.

Cyclic voltammetry experiments were performed in a KCl solution of concentrations from 10^–1^ to 10^–6^ M in a potential range from 0 to 1.2 V at a scan rate of 5 mV s^–1^.

The emission increase onset potential, *E*_EIO_, was obtained as the cross section of both extrapolated linear portions of emission vs applied potential dependence: part of dependence where emission is independent of applied potential and the part where abrupt increase in emission is observed for cathodic polarization - half scans (Figure S2).

FE-ISE, carbon paper, and glassy carbon (0.07 cm^2^) electrodes were also tested by cyclic voltammetry in 0.01 M K_4_[Fe(CN)_6_] dissolved in 0.1 M KCl water solution in the same potential range as above.

Chronoamperometric measurements were carried out for a KCl solution of the concentration as above with sequential pulses at 1.2 V (regeneration–oxidation of POT) and at 0.2 V (signal–reduction of POT) with different pulse duration times. Charge passed in the experiment was calculated by integration of the current over time.

AC impedance spectra were collected over a wide frequency range (0.01–100,000 Hz) at a bias of 0.3 V and amplitude of 50 mV.

## Results and Discussion

The new type of fluoroelectrochemical ion-selective membrane is proposed comprising a tailored composition of POT together with an ionophore (L) and cation–exchanger (R^–^) in a plasticized PVC matrix that result in one layer.

The emission spectra recorded in 0.1 M KCl are shown in Figure S3. Two maxima present in spectra at ca. 670 and 720 nm, with intensity dependent on applied potential, are characteristic of nanostructures of POT^14^ formed in the PVC matrix.^20^ The thickness of the FE-ISE was estimated to be close to 78 μm. The resistance of the prepared sensor, estimated from chronopotentiometric experiments performed in 0.1 M KCl using a cathodic/anodic current equal to 10^–8^ A, was close to 2.5^.^10^5^ Ω (Figure S4A). This experiment shows also almost linear dependence of the potential on time with a small curvature pointing to diffusion limitations across the membrane resulting from transport of either ions or ionophores.

In the case of slow diffusion of the ionophore from the membrane bulk to the membrane/solution interface (where the ionophore interacts with potassium ions entering the membrane), decrease in the free ionophore concentration in the surface layer can occur. Under galvanostatic conditions the surface concentration of ionophore, *c*(0, *t*) can be estimated from eq 1:^23^
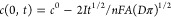
1where *c*^0^ is the free ionophore concentration in the membrane bulk (ca. 0.08 M), *I* is the applied current (1^.^10^–8^ A), *t* is the time (30 s), *D* is the diffusion coefficient of the ionophore in the membrane (2^.^10^–8^ cm^2^/s),^24^ and the other symbols have their usual meaning. The result of calculation shows that ionophore depletion close to the membrane surface, under the above given conditions, is negligibly small (below 0.1%).

An exemplary impedance spectrum of the sensor is shown in Figure S4B. It represents a high-frequency semicircle, suggesting a membrane resistance close to 1.5^.^10^5^ Ω and Warburg impedance behavior for lower frequencies. The slightly lower resistance obtained from this experiment (compared to chronopotentiometry, Figure S4A) may be explained by ion concentration polarization in the membrane under galvanostatic conditions, resulting in apparent membrane resistance increase.^25^ The Warburg impedance confirms diffusional limitations in ion transfer in the membrane suggested by chronopotentiometric experiments (described above). Assuming the determined resistance and current of a range of 10^–8^ A, as used in chronopotentiometry, the estimated ohmic drop in the membrane is in a range of a single millivolt. Therefore, migration effects are of minor significance for ion transfer in the membrane under these conditions and diffusion is the predominant mode of transport.

The electrochemical properties of the FE-ISE are similar to those of typical ISMs, and the presence of POT in the membrane is not resulting in the sensitivity of the layer to solution redox systems ([Fe(CN)_6_]^3–/4–^) (Figure S4C).

The principle of the fluoroelectrochemical approach can be summarized by the following reaction (eq 2) 
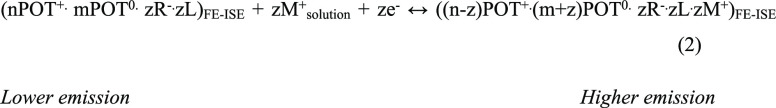
2where M*^+^* is the analyte; e*^–^* is the electron; *n*, *m*, and *z* are stoichiometric coefficients; POT*^+^* represents the oxidized (quenched) polymer backbone and POT^0^ the neutral (emissive) polymer backbone; and the FE-ISE denotes the membrane phase.

Reduction of POT^+^ requires incorporation of cations to compensate charge changes in the membrane, and POT^0^ generated contributes to the recordable increase in emission. The process is reversible: formation of POT^+^ in the membrane requires expulsion of analyte ions and results in emission decrease. The redox process of POT present in the membrane, as shown by eq 2, is dependent on the applied trigger and primary ion concentration, leading to a change in the emission of the system quantitatively corresponding to the electrochemical process occurring. Thus, the herein proposed approach allows translation of the electrical signal, related to selective exchange of primary ions with solution, into a high-sensitivity optical signal.

**Figure 2:**
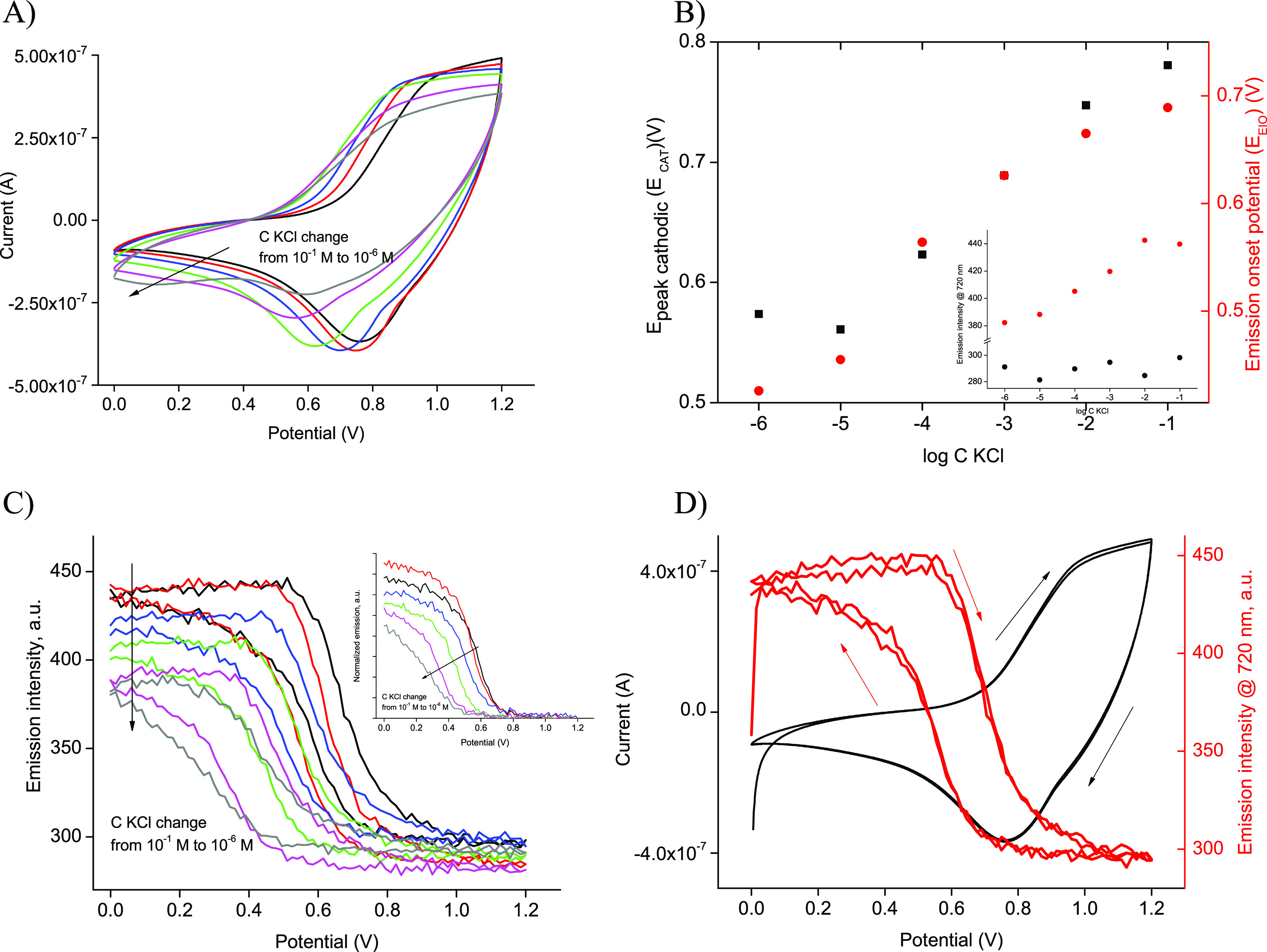
(A) Cyclic voltammograms of (black) 10^–1^ M, (red) 10^–2^ M, (blue) 10^–3^ M, (green) 10^–4^ M, (magenta) 10^–5^ M, and (gray) 10^–6^ M KCl solutions (5 mV/s). (B) Dependence of cathodic peak potential (*E*_CAT_) and emission onset potential (*E*_EIO_) on log *C* KCl; inset: dependence of emission intensity at 720 nm recorded on the logarithm of KCl concentration at (solid red circles) 0 V and (solid black circles) 1.2 V. (C). Emission at 720 nm was recorded during the voltammetric experiment; inset: normalized emission changes for cathodic polarization (half scans). (D) CV and emission intensity change recorded in a 10^–1^ M KCl solution; two consecutive scans are shown.

Cyclic voltammograms (CVs) recorded for FE-ISM in KCl solutions are shown in Figure 2A. Although the FE-ISM was relatively thick compared to that typically used in ion-selective membrane voltammetric experiments, e.g.,^3^ on a cathodic scan, a peak, attributed to cations incorporation to the membrane, was formed at *E*_CAT_. *E*_CAT_ shifts with a decreasing electrolyte concentration to a lower value, with the slope of *E*_CAT_ on logarithm of the KCl concentration (log *C* KCl) close within the range of experimental error to Nernstian 56.4 ± 3.6 mV/dec (for a range of 10^–5^–10^–1^ M, *R*^2^ = 0.988) (Figure 2B). Thus, the herein proposed FE-ISE of nearly 80 μm thickness can be also useful in electrochemical only studies as an alternative to thin, thus prone to deterioration, ion-selective membranes.

Oxidation and reduction of POT in the FE-ISM can be also observed as change in the emission intensity (Figure 2C,D). An initially applied potential results in some increase in emission (Figure 2D); however, the onset of oxidation current increase corresponds to an abrupt decrease in emission intensity due to the decrease in number of neutral polymer backbones in favor of POT^+^ formation. The magnitude of the recorded current and emission signal for 0.1 M KCl decreases for potentials close to or higher than 1 V. On the reverse scan, an initially small increase in emission is observed followed by an abrupt increase for potentials lower than that of the cathodic peak. It should be stressed that both current and emission response were reproducible (Figure 2D). Comparison of emission changes recorded for different KCl concentrations (Figure 2B), clearly confirms the sensitivity of the emission approach to follow changes occurring in response to an applied potential trigger. In cathodic scans, both *E*_CAT_ and emission increase onset potential (*E*_EIO_) are moving toward lower values with a decreasing electrolyte concentration. The linear range of *E*_EIO_ vs log *C* KCl is shifted to lower concentrations compared to *E*_CAT_ dependence, and it covers the range from 10^–2^ to 10^–6^ M, with a slope of 65.0 ± 6.9 mV/dec (*R*^2^ = 0.967). These results clearly show that emission readout offers advantages compared to current signals at low concentrations, which can be attributed to the high sensitivity of the emission approach in general and independence of fluorimetric signals recorded from ohmic drop related to the ion-selective membrane and/or diluted sample solution.

Sample concentration change also affects emission values read at a 0 V (lowest cathodic) potential applied to the FE-ISM. A linear dependence of emission read at 0 V on log *C* KCl was obtained within the range from 10^–5^ to 10^–2^ M (*R*^2^ = 0.991); formation of reduced, emissive POT^0^ is dependent on the electrolyte concentration. On the other hand, at 1.2 V, formation of POT^+^ occurs in the FE-ISM; this process is independent of analyte concentration, and emission recoded at this potential is practically independent of log *C* KCl.

The effect of KCl concentration influence on voltammetric curve peak positions on the potential scale is related with the rate-limiting step (rds) in this process. The rds in this case (as confirmed by EIS results for the low-frequency range showing a Warburg impedance effect in the time domain typical for voltammetry, Figure S4B) is ion diffusion in the membrane. This process is independent of mass transfer phenomena in solution, and thus it is independent of KCl concentration unless the concentration in solution is very low. As expected for a KCl concentration > 10^–4^ M, recorded current magnitude under voltammetric conditions is practically independent of KCl concentration and somewhat decreases for the lower concentrations tested (10^–5^ and 10^–6^ M) (Figure 2A). On the other hand, the process (equilibrium) of ion exchange at the membrane/solution interface is implemented in the series of processes starting with mass transfer in solution and ending with POT reduction and electron flow in the external circuit. Since the membrane potential is dependent on KCl concentration in a Nernstian manner, the concentration-dependent shift of the peak potential is observed (Figure 2B).

**Figure 3:**
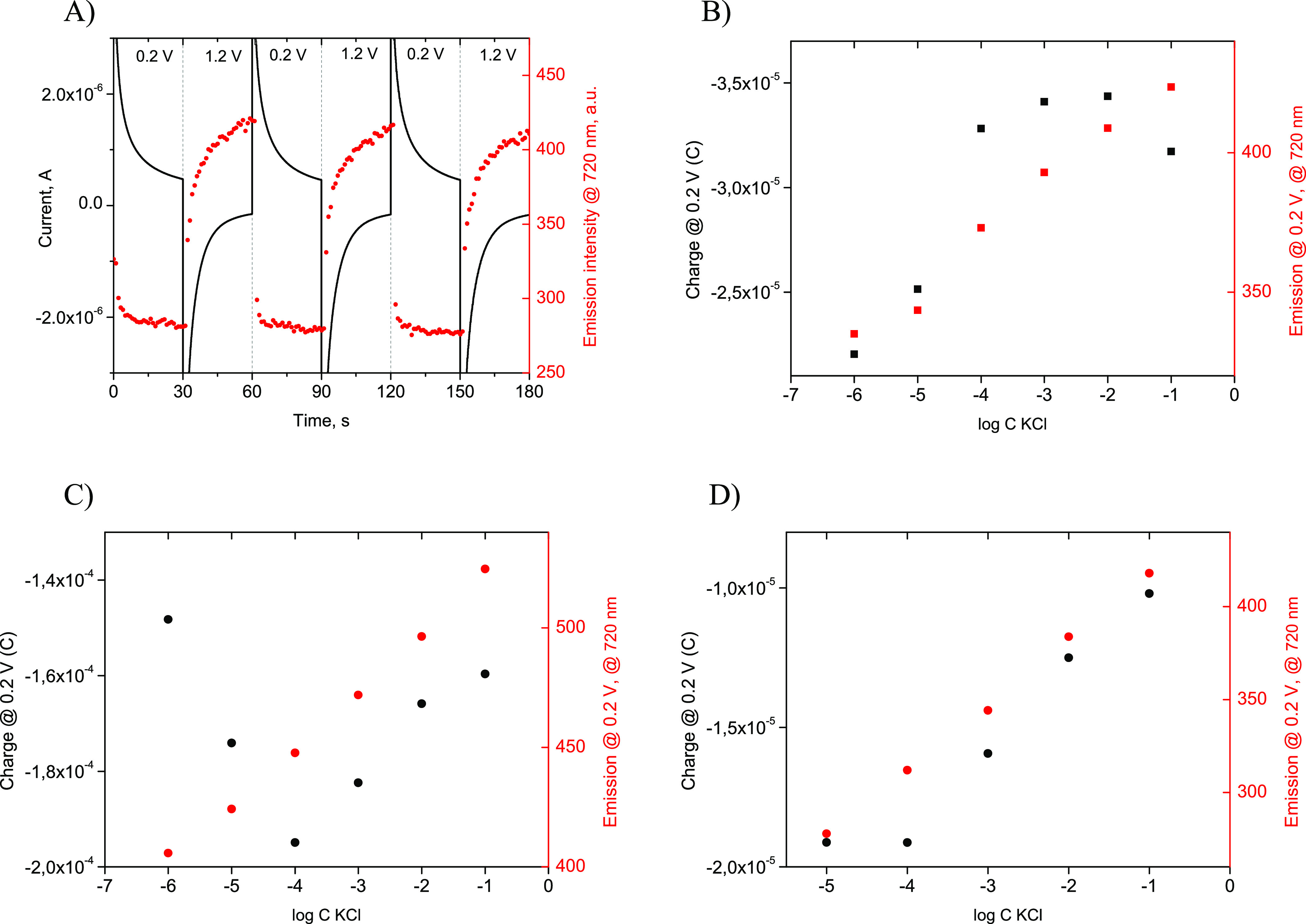
(A) Changes of (black) current and (red) emission recorded in 10^–2^ M KCl solution during consecutive polarization at 0.2 V (signal) and 1.2 V (regeneration) of the sensor. (B–D) Coulometric (black) dependence of charge recorded at 0.2 V vs logarithm of KCl concentration and (red) emission read at a maximum of 720 nm at 0.2 V (B) (solid black/red circles) for 30 s at 0.2 V after a 30 s pulse at 1.2 V, (C) (solid black/red squares) for 180 s at 0.2 V after a 360 s pulse at 1.2 V, and (D) (solid black/red circles) for the thin FE-ISM for 30 s at 0.2 V after a 120 s pulse at 1.2 V.

The role of diffusion in the membrane as the rds can be also confirmed by comparing the recorded reduction charge with the charge needed to completely reduce POT present in the membrane. The experimentally determined charge is only around 1% of the maximal reduction charge of POT, confirming the charge trapping effect for the polymer particles and only slight reduction of the polymer (Figure 3A).

The observed linear dependence of *E*_EIO_ on the logarithm of KCl concentration can be related to voltammetric results showing a similar linear dependence of the cathodic peak (*E*_CAT_) on the logarithm of KCl concentration (Figure 2B). The shift of peak potential, observed at different KCl concentrations, or the potential difference between the applied potential and peak potential, is coupled with charge flow. As shown on the chronopotentiometric curve (Figure S4A), the potential changes almost linearly with time (i.e., it changes almost linearly with flowing charge under galvanostatic conditions). Since the charge is practically linearly dependent on potential and the potential responds to KCl concentration in a Nernstian manner, the reduction charge of POT will be also linearly dependent on the logarithm of KCl concentration.

Reduction of POT results in formation of a neutral form of the polymer, characterized by emission; thus, in the absence of side faradaic reactions, the fluorimetric signal will be a linear function of charge. Taking all the abovementioned relations, KCl concentration, potential, charge, and emission intensity, the fluorimetric signal is expected to linearly depend on the logarithm of electrolyte concentration, as observed experimentally.

The fluoroelectrochemical ion-selective membrane was proven to be selective both in electrochemical and optical mode (Figure S5). Figure S5A shows CV and corresponding emission changes at 720 nm recorded in NaCl–model interferent–solutions, whereas Figure S5B–D presents CV and emission changes read at 720 nm recoded in 10^–2^ M KCl, NaCl, CaCl_2_, or MgCl_2_. These results clearly confirm that in the presence of interfering ions, *E*_CAT_ values are shifted to lower potentials as expected^3^ for hindered access of interfering ions to the FE-ISE during reduction. On the other hand, changes of emission at 720 nm, accompanying the recorded CVs in interfering ions, were significantly smaller for Na^+^, Ca^2+^, or Mg^2+^ compared to those observed in potassium ion solutions (Figure S5B). *E*_CAT_, *E*_EIO_, and emission (at 720 nm) recorded at 0 V or at 1.2 V were only slightly dependent on changes of the logarithm of concentration of interfering ions (Figure S5E,F) compared to the effect of KCl concentration change shown in Figure 2B. These results clearly confirm the high selectivity of the herein proposed FE-ISE, both using electrochemical and optical readouts of generated signals.

The coulometric readout of ISE^4^ requires a redox reaction of POT, opening the possibility of emission signal recording. Figure 3A shows exemplary changes of current recorded for repeatedly applied potential corresponding to reduction–signal generation (0.2 V) and oxidation–regeneration of FE-ISE (1.2 V) and corresponding changes of emission read at 720 nm. Clearly, emission signal stabilization is quicker, especially at lower potentials, where the process is controlled by incorporation of analyte cations from the solution to the membrane phase. For regeneration of the membrane at higher potentials, repulsion of ions from the FE-ISE, as expected, is a slower process. It should be stressed that repeated polarization of the sensor at 0.2 and 1.2 V results in similar, within the range of experimental error, changes and ultimate values of current/emission recorded. Figure 3B–D shows dependence of charge (integrated current recorded at 0.2 V) and emission value plotted as a function of log *C* KCl. Figure 3B shows that the change of concentration does not affect significantly the charges recorded within the range from 10^–1^ to 10^–4^ M; however, for lower concentrations, pronounced changes are observed. On the other hand, the emission signal recorded was linearly dependent on log *C* KCl within the whole tested range from 10^–6^ to 10^–1^ M (*R*^2^ = 0.984). This clearly shows that the emission signal advantageously allows significant extension of the linear response range. This effect is ascribed to the high sensitivity of POT emission changes for alteration of the polymer redox state. Extending the time of potential pulses resulted in somewhat different response patterns and higher sensitivity both in coulometric and emission modes (Figure 3C); however, it did not affect the linear range of emission dependencies—linear responses were obtained within the whole range 10^–6^–10^–1^ M KCl (*R*^2^ = 0.996). The broader linear range of signal vs logarithm of concentration dependence of the emission approach was proven for a thinner FE-ISE (Figure 3D). The emission readout resulted in a linear relation of emission on log *C* KCl within a range of 10^–5^–10^–1^ M (*R*^2^ = 0.999) compared to the linear relation of charge vs log *C* KCl within the order of magnitude shorter range from 10^–4^ to 10^–1^ M KCl (*R*^2^ = 0.994) (Figure 3D). The observed difference in dependence of charge on the logarithm of KCl concentration, for thinner and thicker membranes, may be related to the shape of the chronoamperometric curve with a relatively high current just after potential pulse application, which may lead to some errors in charge calculation (integration).

In conclusion, the novel signal transduction of ion-selective sensors operating under an electrochemical trigger is presented. We have demonstrated for the first time the application of emission changes of the dye embedded within the membrane as an alternative signal for the electrochemical signal under conditions of voltammetric or coulometric experiments. The emission correlates with electrochemical responses observed under non-zero-current conditions, offering lower detection limits and broader linear response ranges under coulometric conditions. The new emission readout principle can be extended to a wide range of ion-selective systems under various non-zero-current electrochemistry conditions.

## Supporting Information Available

Scheme of electrode used, emission increase onset potential determination method, emission spectra of the fluoroelectrochemical ion-selective membrane recorded in an open circuit and while applying oxidizing or reducing potentials and corresponding chronoamperometric dependencies, chronopotentiometric studies and impedance spectra of fluoroelectrochemical ion-selective membranes applied on glassy carbon or carbon paper substrates, effect of interfering ions presence on cyclic voltammograms, and emission changes recorded for FE-ISE (PDF)
