# Supplementary material for: Emission Intensity Readout of Ion-Selective Electrodes Operating under an Electrochemical Trigger
Source: Anal Chem. 2021 Jul 15;93(29):10084–9. doi: 10.1021/acs.analchem.1c00857 (PMC8382224; doi:10.1021/acs.analchem.1c00857)
Supplement: Supplementary file 1 — ac1c00857_si_001.pdf [file ac1c00857_si_001.pdf]

Supporting information

Emission intensity readout of ion-selective electrodes operating under electrochemical trigger

Katarzyna Węgrzyn, Justyna Kalisz, Emilia Stelmach, Krzysztof Maksymiuk, Agata Michalska

Faculty of Chemistry, University of Warsaw, Pasteura 1, 02-093 Warsaw, Poland
* agatam@chem.uw.edu.pl, +48 22 56 22 331

Table of Content

1. Scheme of electrode used

2. Emission spectra of FE-ISE membrane recorded in open circuit, as well as while applying oxidizing or reducing potential

3. Chronoamperometric dependencies corresponding to emission spectra presented above - applying oxidizing or reducing potential to FE-ISE membrane sensor.

4. Chronopotentiometric studies and impedance spectra of FE-ISE membranes applied on glassy carbon or carbon paper substrate,

5. Effect of interfering ions presence on cyclic voltammograms and emission changes recorded for FE-ISE

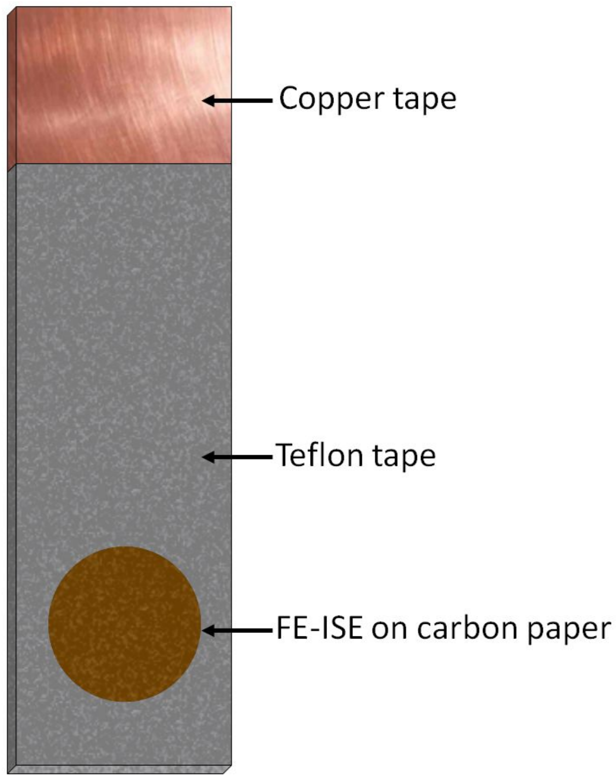

Figure S1. Scheme of electrode used.

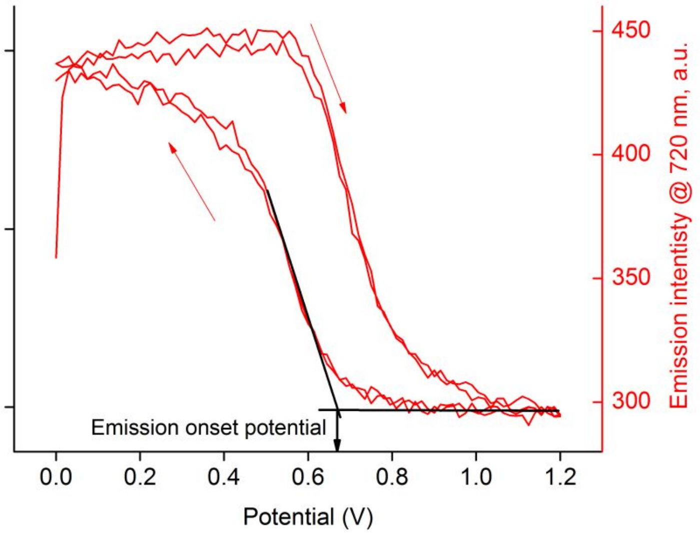

Figure S2. Graphical representation of emission increase onset potential, E_{EIO}, determination method, using emission vs applied potential dependence.

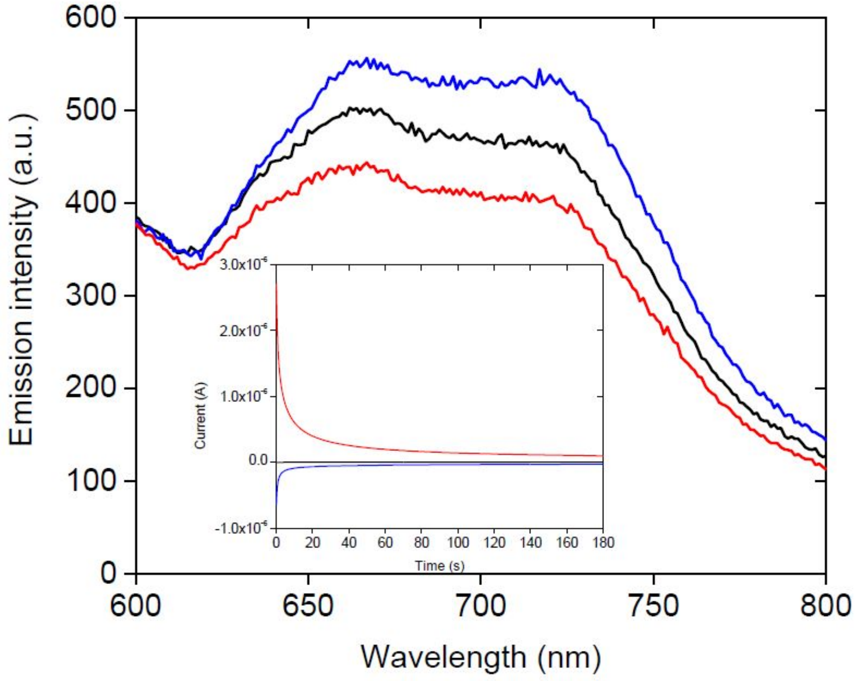

Figure S3. Emission spectra recorded for ion-selective membrane containing POT in 0.1 M KCl recorded under (black line) open circuit conditions, as well as recorded while applying (red) oxidizing (1.2 V)/ (blue) reducing (0.2 V) potential for 120 s before and during emission scan. Inset: complementary chronoamperometric dependencies.

A)

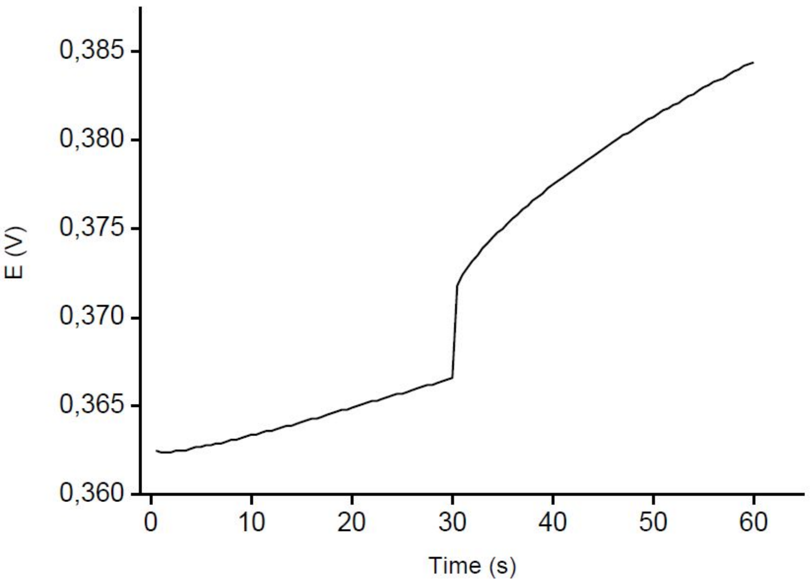
0,385
0,380
0,375
0,370
0,365
0,360
E (V)
0
10
20
30
40
50
60
Time (s)

B)

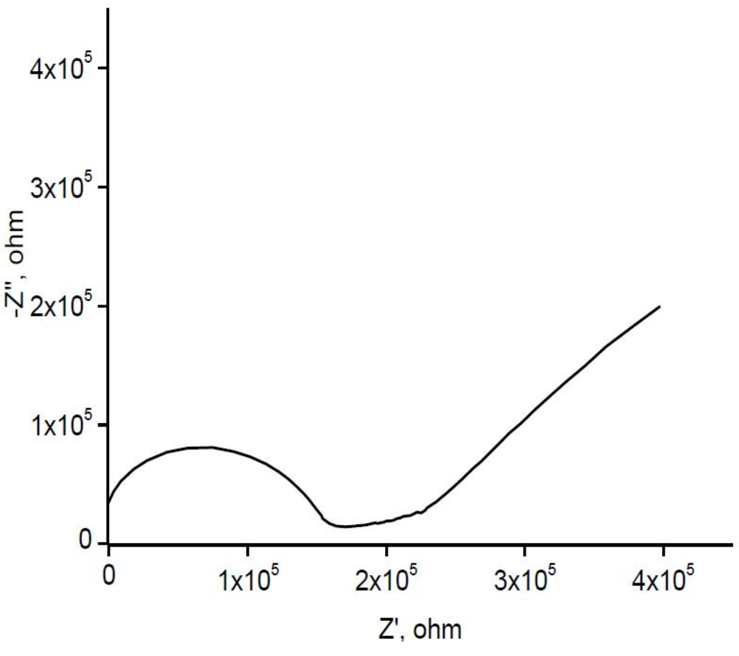
4x10^5
3x10^5
2x10^5
1x10^5
0
-Z'', ohm
0
1x10^5
2x10^5
3x10^5
4x10^5
Z', ohm

C)

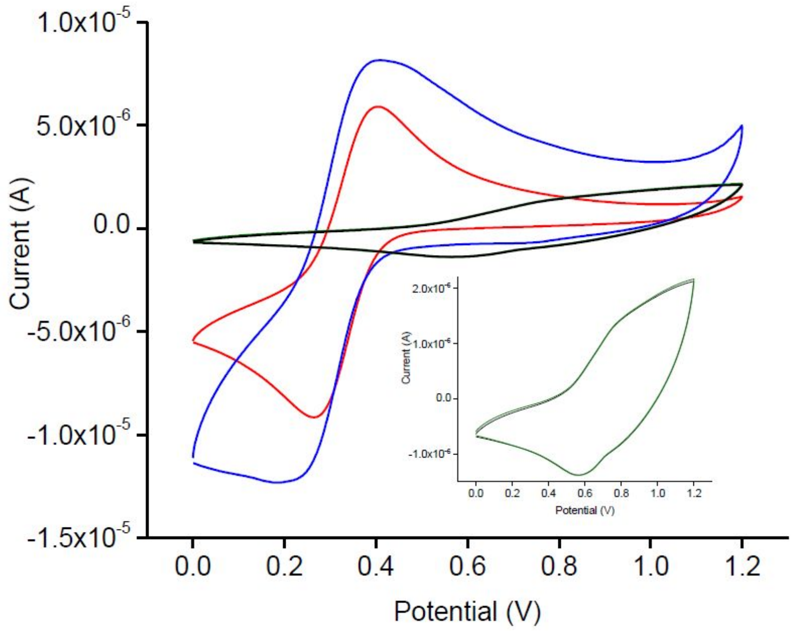

Figure S4. Electrochemical studies of FE-ISE membranes: A) Chronopotentiometry applying current equal to $1{\cdot}10^{-8}$ A in 0.1 M KCl, B) Impedance spectra of tested sensors recorded in 0.1 M KCl, at potential 0.3 V, C) cyclic voltammograms (scan rate 50 mV/s) recorded for: (blue line) support (unmodified) carbon paper, (red line) glassy carbon electrode, (black line) as prepared sensors with carbon paper support and FE-ISE membrane in solution containing 10 mM $K_4Fe(CN)_6$ in the presence of 0.1 M KCl, for comparison trace recorded for as prepared sensors with carbon paper support and FE-ISE membrane in 0.1 M KCl – in the absence of redox couple is also shown (green line) – this trace overlaps with the black line as shown on inset.

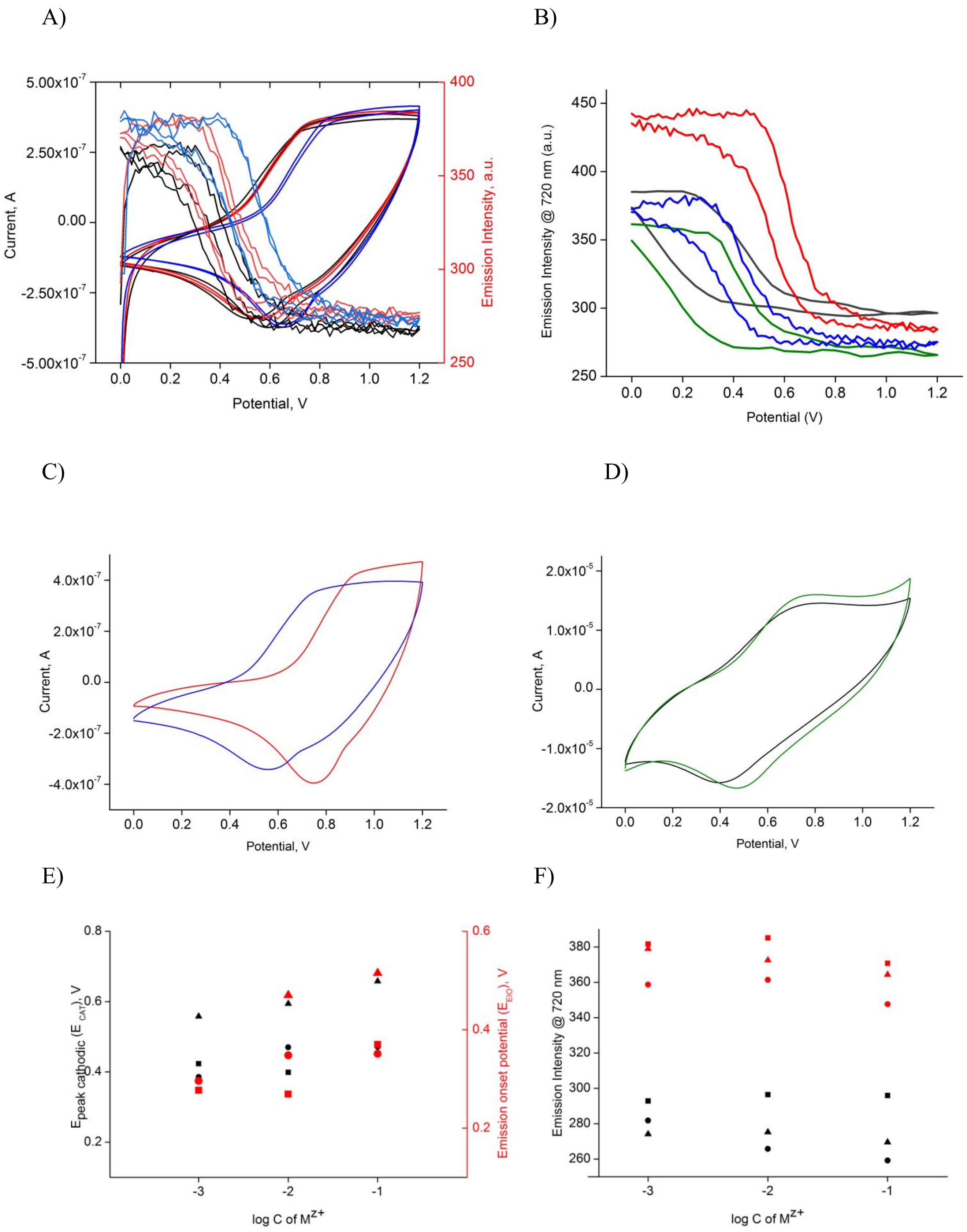

Figure S5. A) Cyclic voltammograms of (black) 10^{-1} M, (red) 10^{-2} M, (blue) 10^{-3} M, 5 mV/s recorded in NaCl and emission read at 720 nm recorded during CV experiments. B) Emission

changes recorded during and cyclic voltammograms experiments performed in 10^{-2} M solutions of (red) KCl, (black) $CaCl_2$, (blue) NaCl, (green) $MgCl_{2:}$ B) scan rate 5 mV for KCl an NaCl, and C) 50 mV/s for $CaCl_2$ and $MgCl_2$.) , C) and D) cyclic voltammograms. E) Dependence of (black symbols) cathodic peak potential (E_{CAT}) and (red symbols) emission onset potential (E_{EIO}) on log C of model interferents tested: (■/■) $CaCl_2$, (●/●)$MgCl_2$, (▲/▲) NaCl. F) Dependence of emission recorded on logarithm of (■/■) $CaCl_2$, (●/●)$MgCl_2$, (▲/▲) NaCl concentration at (red symbols) 0 V and (black symbols) 1.2 V.
